# An Application of a LPWAN for Upgrading Proximal Soil Sensing Systems

**DOI:** 10.3390/s22124333

**Published:** 2022-06-08

**Authors:** Yonghui Tu, Haoye Tang, Wenyou Hu

**Affiliations:** 1 State Key Laboratory of Soil and Sustainable Agriculture, Institute of Soil Science, Chinese Academy of Sciences, Nanjing 210008, China; yhtu@issas.ac.cn (Y.T.); hytang@issas.ac.cn (H.T.); 2 Key Laboratory of Soil Environment and Pollution Remediation, Institute of Soil Science, Chinese Academy of Sciences, Nanjing 210008, China

**Keywords:** IoT, LPWAN, proximal soil sensor device, conventional communication methods, ultralow power consumption, long-distance transmission, economic value, inventoried sensor devices

## Abstract

In recent years, the Internet of Things (IoT), based on low-power wide-area network (LPWAN) wireless communication technology, has developed rapidly. On the one hand, the IoT makes it possible to conduct low-cost, low-power, wide-coverage, and real-time soil monitoring in fields. On the other hand, many proximal soil sensor devices designed based on conventional communication methods that are stored in an inventory face elimination. Considering the idea of saving resources and costs, this paper applied LPWAN technology to an inventoried proximal soil sensor device, by designing an attachment hardware system (AHS) and realizing technical upgrades. The results of the experimental tests proved that the sensor device, after upgrading, could work for several years with only a battery power supply, and the effective wireless communication coverage was nearly 1 km in a typical suburban farming environment. Therefore, the new device not only retained the original mature sensing technology of the sensor device, but also exhibited ultralow power consumption and long-distance transmission, which are advantages of the LPWAN; gave full play to the application value and economic value of the devices stored in inventory; and saved resources and costs. The proposed approach also provides a reference for applying LPWAN technology to a wider range of inventoried sensor devices for technical upgrading.

## 1. Introduction

Soil is an important natural resource and the most critical material basis for agricultural production. The acquisition and analysis of data related to soil moisture, salt, pH, and other physicochemical properties is an important basis for land resource utilization and agricultural production activities. Conventional soil sampling and laboratory analyses have long sampling and analysis cycles and high labor costs; therefore, various proximal soil sensor devices and data acquisition systems have been widely used in fields [1,2]. Proximal soil sensing mainly refers to the use of field sensors to acquire information proximal to the ground or in the soil. This concept was first proposed by Viscarra Rossel and McBratney in 1998 [3] and further developed in 2010 [4]. At present, various proximal ground sensor devices based on different working principles have been developed. For example, the EM38 conductivity meter developed by Geonics Inc. (Mississauga, ON, Canada) is an instrument used to obtain soil comprehensive apparent electrical conductivity (ECa) based on the principle of electromagnetic induction. Myers et al. used this instrument to combine conductivity data from the soil surface and soil profiles for high-resolution ECa soil digital mapping [5]. Besson et al. used MUCEP (multi-continuous electrical profiling) to measure soil resistance coefficient and monitor the temporal and spatial changes in soil moisture at the field scale [6]. Electrochemical sensors based on ion selective electrodes (ISEs) and ion sensitive field effect transistors (ISFETs) are mainly used for the determination of soil pH and nitrate and potassium ion concentrations. Adsett and Thottan designed a real-time automatic nitrate content measurement system using ISFETs and a nitrate detector [7]. Similar instruments include the pH meters produced by Veris Technologies, Inc. (Salina, KS, USA) and Spectrum Technologies, Inc. (Aurora, IL, USA). These proximal soil sensor devices or systems are usually deployed in field environments and connected to data acquisition devices with RS-485, RS-232, or SDI-12 cables, and manually obtain data on-site. In practical applications, such systems face the problems of limited bearing capacity, cumbersome wiring, high operation costs, and inconvenient installation and maintenance. With the rapid development of information technology, the IoT has been widely used in various industries, promoting their rapid development and extension. Low-power wide-area networks (LPWANs) form one of the main hotspots of IoT access technology [8]. Compared with conventional wired communication technologies (such as RS-485 and SDI-12), mobile cellular technologies (such as 2G, 3G, 4G, etc.), and short-range wireless communication technologies (such as Bluetooth, ZigBee, etc.), an LPWAN has the advantages of low cost, low power consumption, wide coverage, and strong connection [9], and can effectively achieve the application of a proximal soil sensing system. An LPWAN is an important technical support tool for promoting the transformation from conventional laboratory-based physicochemical soil analyses to field-based measurements. Vu et al. designed an automatic irrigation system for greenhouses based on LoRa technology [10]. Rachmani et al. designed an IoT monitoring system based on LoRa technology for a starfruit plantation [11]. Co et al. designed and developed the hardware and software components of a wireless sensor network (WSN) for soil monitoring [12]. In these applications, the LoRa communication module is usually independent of the sensor device, but the sensor device is still based on the conventional application design, and its power requirements cannot be met by a long-term battery power supply. Thus, the deployment of the system is troublesome, and a lot of maintenance work is required in the later stages of use.

To meet the needs of long-term field work, and considering the limitations of sensor battery power supply, LPWAN technology needs to be integrated and applied in proximal soil sensor devices. However, such devices generally need to be redesigned at a high cost, and this leads to the elimination of the previous generation of inventoried devices, due to their outdated technology. At the same time, the redesigned sensor not only needs to have its communication function tested, but also requires more practice to verify its sensing technology [13]. Therefore, this paper selected an inventoried soil moisture sensor based on an RS-485 interface as the research object; designed and adapted the attachment hardware system (AHS), according to its electrical specifications and communication protocol, integrated LPWAN technology; and realized the technological upgrade of the sensor, so that it not only retained the function and performance of the original sensor, but also had the attributes of ultralow power consumption and long-distance transmission, while supporting long-term battery power supply, easy deployment, and simple management. At the same time, the elimination of an inventoried device due to the application of new technology was avoided, and resources and costs were saved, because the design was based on the inventoried device.

The main contributions of this paper are as follows:

1. This paper put forward a new idea for applying the emerging LPWAN technology in proximal soil sensing systems and carried out engineering practice.
2. Instead of directly eliminating the inventoried proximal soil sensor device with outdated technology, this paper upgraded it by designing an AHS; the new device not only retained the original mature sensing technology of the sensor device, but also exhibited ultralow power consumption and long-distance transmission. In addition, this paper gave full play to the application value and economic value of the devices stored in the inventory.
3. The proposed approach also provides a reference for applying LPWAN technology to a wider range of inventoried sensor devices for technical upgrading.

The rest of this paper is organized as follows: Section 2 presents the overall architecture design of the system. Section 3 describes the hardware design of the AHS. Section 4 introduces the software design of the system. Section 5 tests and analyzes the sensor device, after loading the AHS, and also discusses the relevant factors affecting the communication quality. The last section summarizes this paper and discusses its significance.

## 2. Design of the System Architecture

**Figure 1:**
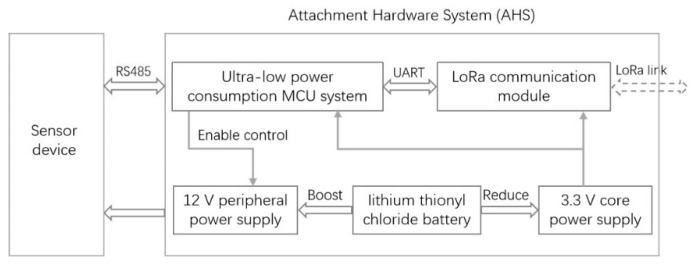
Overall structure of the system.

The AHS, which was designed to adapt inventoried sensor devices, mainly included an ultralow-power MCU system, a communication module, and a power module. The overall architecture is shown in Figure 1. The AHS took the ultralow-power MCU system as its core, and enabled and controlled the boost chip to turn the working power supply of the sensor device on or off. It obtained the data acquired by the sensor device or configured its relevant parameters by adapting the 485 interface communication protocol; connected and controlled its communication module through UART; and exchanged data with the server through ultralow power wireless transmission, which included uploading the data acquired by the sensor device and receiving the control command parameters sent by the server to control the workflow of the system.

## 3. Hardware Design of the AHS

### 3.1. Proximal Soil Sensor Device and Power Supply

**Figure 2:**
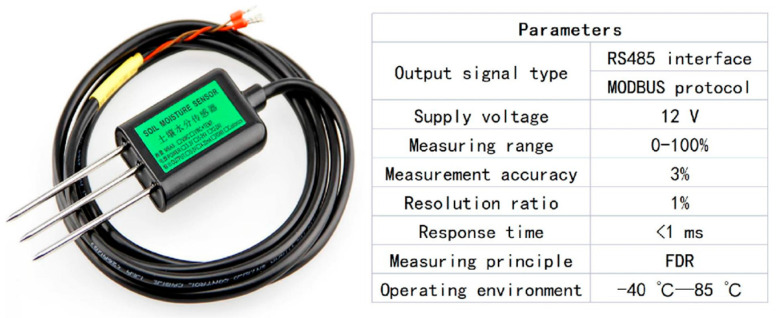
Soil moisture sensor.

Soil moisture is not only an important part of soil fertility and an important factor affecting plant growth and development, but it is also an important parameter for studying agricultural drought and crop drought. Therefore, data acquisition devices and systems based on various soil moisture sensors have been widely used [14]. In this paper, a commercial soil moisture sensor was used as the research object and was taken as the sensing module of the AHS. Its accuracy and reliability have been tested in practice and in the market for a long time. The volumetric moisture content of the soil was measured with an RS-485 standard communication interface, with a working voltage of 12 V and a response time of less than 1 s. Under the condition of no external load, the maximum working current was less than 25 mA, and the average was no more than 10 mA. More parameters are shown in Figure 2.

The system power supply adopted a lithium thionyl chloride battery with an output voltage of 3.6 V and a battery capacity of 3500 mAh. It has the characteristics of high energy density, long service life, and excellent low-temperature performance. It is especially suitable for all-weather battery-based power supply devices in the field [15]. To simplify the hardware structure and facilitate application deployment, the system adopted a single-battery global power supply and an efficient power management scheme. The sensor device adopted a 12 V DC power supply, and the MCU, flash chip, RS-485 transceiver, and other chips adopted 3.3 V power supplies. Therefore, the battery voltage was boosted to 12 V in the circuit hardware, to supply power to the sensor. A stable 3.3 V was output by the multichannel linear voltage regulator to supply power to the chips, in which the main controller (3.3 V) and the peripheral circuit (3.3 V) were independently supplied to eliminate the interaction between loads.

### 3.2. Ultralow-Power MCU System

MCUs typically use CMOS technology, and their power consumption mainly includes static power consumption and dynamic power consumption. Static power consumption mainly consists of the energy consumed by transistors, which is almost constant, most of the time. Dynamic power consumption includes switching power consumption, short-circuit power consumption, and burr power consumption. In general, especially when working at a high frequency, dynamic power consumption plays a major role, which can be approximately expressed as the following Equation (1) [16]:

(1) $$P=C_{L}\times V_{DD}^{2}\times f$$

where $C_{L}$ is the load capacitance, $V_{DD}$ is the supply voltage, and f is the clock frequency. The total power consumption is the sum of the static power consumption and dynamic power consumption. Therefore, to reduce the total power consumption, we can reduce the size of the MCU chip or the number of transistors; reducing the MCU supply voltage can reduce power consumption at the square level and reduce the clock frequency to just meet the application needs. In addition, a reasonable choice of working mode, such as entering sleep mode after working at full speed for a very short time, can also greatly save energy [17,18,19].

**Figure 3:**
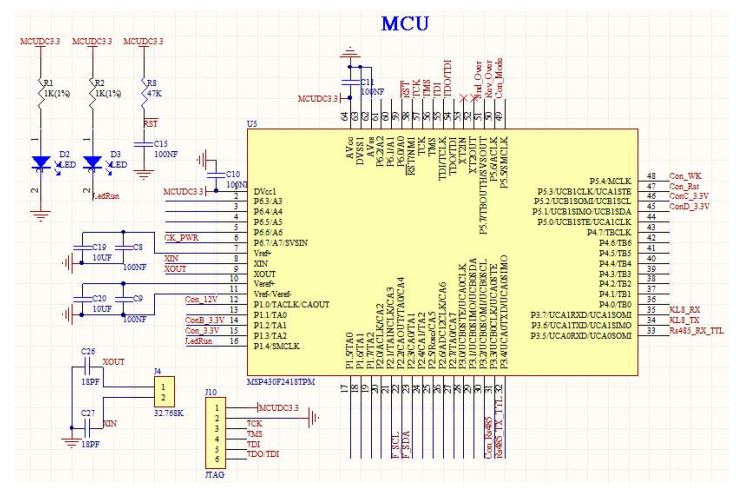
Minimum ultralow-power MCU system.

In this paper, the ultralow-power MCU adopted the MSP430 series, which was specially designed for battery-powered devices in field environments [20]. It adopted a low-power supply voltage of 1.8–3.6 V. When operating under the clock condition of 1 MHz, the power consumption in active mode was only approximately 280 μA, in standby mode it was approximately 1.6 μA, and the minimum power consumption in RAM hold mode was only 0.1 μA. In addition, the MSP430 integrated rich on-chip resources and had multiple interrupt sources, which could be arbitrarily nested and used in a flexible and convenient manner. When the system was in a low-power state, the wake-up interrupt took only 5 μs. The minimum ultralow-power MCU system of the AHS is shown in Figure 3.

### 3.3. Communication Module Based on LoRa

LPWANs have attracted extensive attention, mainly because they can provide affordable connections for low-power devices distributed in very large geographical areas. When realizing the vision of the IoT, LPWAN technologies complement and sometimes even replace conventional wired communication and cellular and short-range wireless technologies, in terms of their performance for various emerging smart city and machine-to-machine applications [21]. Sigfox, LoRa, and NB-IoT are the three leading LPWAN technologies that compete for large-scale IoT deployment, and they have different characteristics that affect the performance of IoT solutions; device connectivity, information delay, and even device battery life [22]. Some of their key characteristics are shown in Table 1.

LoRa has the characteristics of long-distance and low power consumption, which can prolong the battery life. It uses the unlicensed Sub-1GHz ISM bands and does not need to pay additional licensing fees. In addition, LoRa can adapt the data rate and allow private networks, while Sigfox and NB-IoT cannot [23]. LoRa, as a representative LPWAN technology, has emerged as an attractive communication platform for the IoT [24,25]. Therefore, in this paper, the mature commercial LoRa module, which was designed based on SemTech sx1278 (Camarillo, CA, USA), was used as the communication module of the AHS, with an adjustable transmission power and a maximum transmission power of 20 dBm; it supported remote wake-up in sleep mode and adopted advanced channel coding technology. Its receiving sensitivity could reach −142 dBm, enabling it to realize long-distance communication under ultralow power consumption. The LoRa gateway was designed based on a sx1301 transceiver controller. The gateway has a higher receiving sensitivity than other technologies, its sight distance coverage radius can reach 5 km, it includes eight receiver channels and one transmission channel (among which 8 receiver channels can receive data simultaneously), and it supports up to 10,000 LoRa terminals, which are convenient for building a massive connection network. It can also support LTE (4G/3G/2G), connect to servers without wiring, and adapt to the multiple access modes of PAAS platforms, such as MQTT, TCP, and Modbus [26].

## 4. Software Design

### 4.1. Software Design of the MCU

To reduce power consumption, in addition to selecting low-power devices in the hardware design, the key modules also adopted an efficient power management algorithm in the software design of the MCU. The working voltage of the soil moisture sensor device, which was one of the main energy consumption components of the system, was high. However, in practical applications, the sensor device does not need to work continuously for a long time. In a data acquisition and transmission cycle, it would be idle most of the time. Therefore, a 12 V power supply that enabled control was designed for the hardware. When the sensor device worked and effectively outputted data, the MCU controlled the MOS tube to be in the cut-off state and turned off the power supply of the sensor device to avoid continuous power consumption after data acquisition. The LoRa RF communication module was also a main energy-consuming unit, and the working currents corresponding to 5 dB and 20 dB transmission powers were 75 ma and 130 mA, respectively. When the module did not need to work, the MCU would put it to sleep.

**Figure 4:**
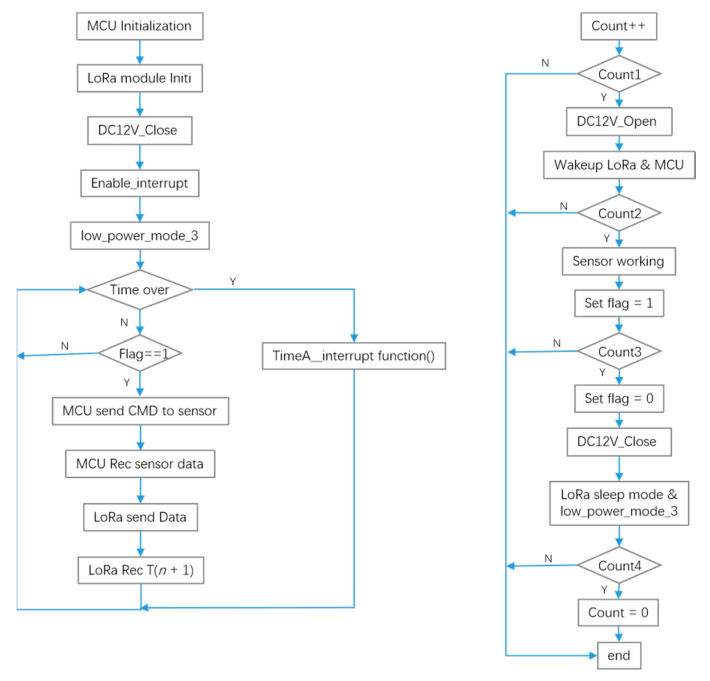
Software flow diagram and timer interrupt function.

The working flow of the MCU software is shown in Figure 4. After the initialization of the MCU and each module, the MCU controlled the MOS tube to be in the cut-off state, turned off the sensor power supply to reduce energy consumption, enabled the MCU to interrupt, and then entered the low power consumption mode. The timer interrupt function was used to realize the acquisition of sensor data, and the MCU timer could automatically overload the time constant, thereby accurately controlling each module to complete different tasks during different timer cycles (250 ms).

### 4.2. Server Design

**Figure 5:**
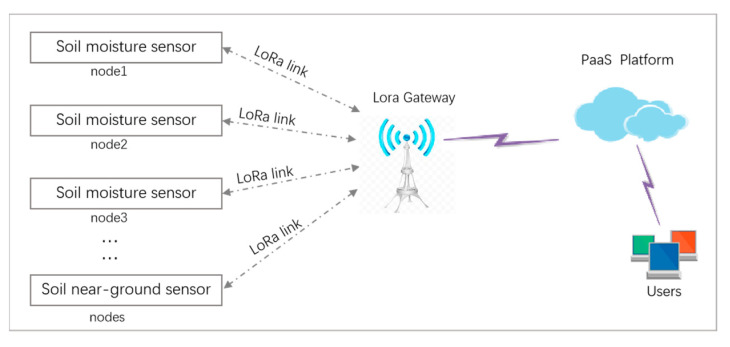
Overall system network structure.

The data acquired by the sensor device were finally transmitted to the server for storage and user access. The LPWAN system was composed of a sensor device loaded with the AHS as a node. Its network structure diagram is shown in Figure 5.

**Figure 6:**
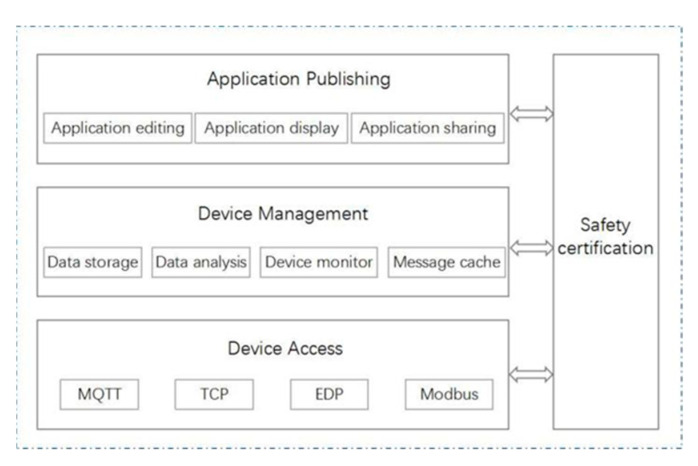
IoT platform architecture based on a PaaS.

Although a server built based on a private cloud can control all resources, such as computing and storage resources, and enjoy exclusive use rights, it also faces high design, installation, deployment, and upgrading costs, and cannot meet the connection requirements of an increasing number of sensor devices and the management requirements of data for multiple future applications [27]. Therefore, this paper used the operator’s IoT platform (OneNet) based on a PAAS as the service end, which was efficient, stable, and safe; could adapt to a variety of network environments and common transmission protocols; provided a fast access scheme, a management service, and data storage capacity for terminal devices; facilitated data storage and querying; and had flexible on-demand payments and controllable costs [28]. Its architecture is shown in Figure 6.

**Figure 7:**
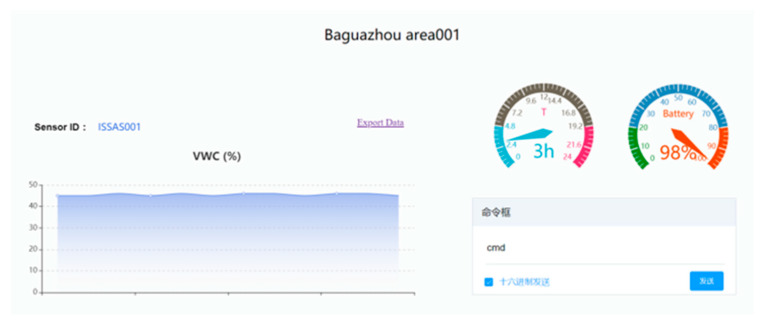
Server application interface.

In this paper, the sensor node used TCP-based transparent communication to access the IoT platform of the server. We customized the protocol content, wrote the protocol analysis script in the Lua scripting language, and uploaded the analysis script to complete the protocol analysis. The application interface is shown in Figure 7.

## 5. System Test and Analysis

### 5.1. Actual Energy Consumption Test and Analysis

**Figure 8:**
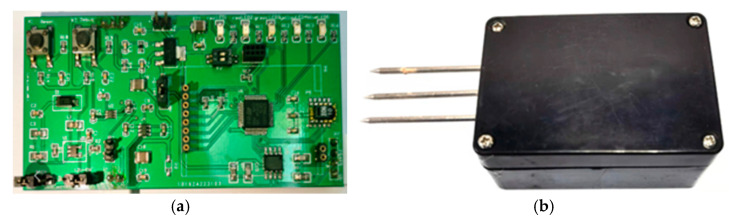
Hardware of the system. (**a**) Physical object of the AHS; (**b**) Soil moisture sensor after loading the AHS.

The physical object of the AHS and the encapsulated soil moisture sensor loaded with the AHS are shown in Figure 8.

Energy consumption is a major problem for battery-powered devices. Once the power is exhausted, the device will “strike”. Although the system minimized the energy consumption of device selection and algorithm design at the beginning of the design process, there needed to be a gap between the actual energy consumption and the theoretical value [29]. To analyze the actual energy consumption performance of the system, the energy consumption of the sensor device after loading the AHS was tested by connecting a high-precision multimeter in series in the system; the real current of the system in each state was measured, and its single service life was estimated according to the battery capacity. When designing the hardware circuit of the AHS system, a special current test interface was reserved so that the jumper would be used for the short circuit during operation, and the multimeter could be directly connected in series during measurement. In this experiment, the DC micro-ampere mode of a Fluke (18B+) multimeter was used. The interrupt timing cycle was set to 250 ms, the system was initialized within the initial 2 s, and the MCU entered the low power consumption mode after configuring the LoRa module. At the 4th second, the MCU exited the low power consumption mode, the ADC started sampling the battery voltage, and the MCU entered low power consumption mode again after sampling. At the 10th second (COUNT1), the MCU turned on the 12 V power supply of the sensor, the MCU exited low power consumption mode and woke up the LoRa. At the 13th second (COUNT2), the sensor started working, the MCU acquired the sensor data, and LoRa started sending and receiving data. At 15 s (COUNT3), the sensor power supply was forcibly turned off, the MCU entered low power consumption mode, and the LoRa entered sleep mode.

The energy consumption of each main state of the system is shown in Table 2. If the sampling period T was 2 h, i.e., 2 × 3600 s, the energy consumption in one cycle can be expressed as E0 = 2.99 J. If the battery capacity P1 = 3500 mAh, then the single battery energy was E = P1 × 3.6 V = 45,360 J, and the battery life was 2 × E/E0 = 30,340 h; approximately 3.46 years. When working with ultralow power consumption, if effective data were acquired every two hours, a single-battery power supply could work for more than 3 years without considering natural attenuation, thus meeting the requirements of general applications. Additionally, flame-retardant epoxy resin could be used for integral molding and pouring; this would make the system more compact as a whole, with high mechanical strength, strong heat resistance, and easy deployment, as well as being maintenance-free, waterproof, and anti-corrosion. 

### 5.2. Channel Characteristics and Gateway Capacity Analysis

The key parameter settings of the node are shown in Table 3. When setting the parameters of radio device, on the basis of meeting the radio management specifications, we optimized the LoRa modulation and demodulation technology through designing the key parameters, such as modulation spread factor, modulation bandwidth, and error correction coding rate, to make the system reach an optimal state, as far as possible [30,31]. The spread spectrum LoRa modulation is performed by representing each bit of the payload information using multiple chips of information. The rate at which the spread information is sent is referred to as the symbol rate; while, the ratio between the nominal symbol rate and chip rate is the spreading factor and represents the number of symbols sent per bit of information. Spread spectrum transmission can reduce the bit error rate; that is, the SNR, as shown in Table 4. Under the condition of a negative signal-to-noise ratio, the signal can be received normally, which improves the sensitivity, link budget, and coverage of the LoRa receiver, but reduces the actual data that can be transmitted under the condition of the same amount of data [32]. Therefore, the larger the spread spectrum factor, the smaller the number rate (bit rate) of the transmitted data. In this paper, we set the spreading factor (SF) as 12 to maximize the signal coverage, under the condition of meeting the transmission rate. The LoRa modem employs cyclic error coding to perform forward error detection and correction. Such error coding incurs a transmission overhead, but it can further improve the robustness of the link. Therefore, we set the coding rate (CR) as 4/5. An increase in signal bandwidth (BW) permits the use of a higher effective data rate; thus, reducing transmission time at the expense of a reduced sensitivity improvement [33]. Apparently, there are regulatory constraints in most countries on the permissible occupied bandwidth. As it is stipulated in China that the power in 470~−510 mHz frequency band shall not exceed 50 mW (17 dBm (ERP)) and the occupied bandwidth shall not exceed 200k [34], we set the bandwidth to 125 k; considering the cable loss and air path loss, we set the transmission power of the node to 20 dBm. In short, these parameters were closely related to the range and robustness of radio communication links. Changing the BW, SF, and CR would change the link budget and transmission time. It was necessary to have a trade-off between battery life and distance.

**Figure 9:**
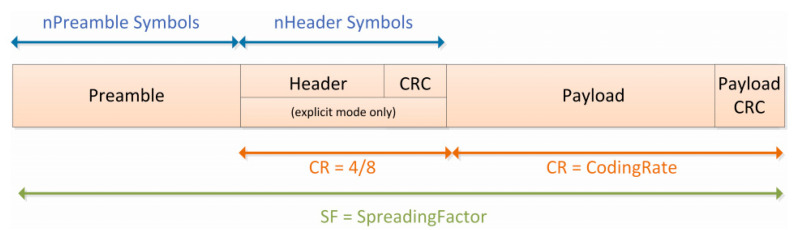
LoRa packet structure.

For large-scale LoRa connection applications, gateway capacity is an important characteristic [35,36], especially in a typical suburban farming environment; and whether the gateway is sufficient for the determined number of nodes is an important concern. In the same application scenario, for a certain gateway, the maximum number of packets that can be received per day is also determined. However, different packet forms and sending rates will change the total number of packets. The LoRa standard data frame format is shown in Figure 9.

The data frame includes a preamble byte, a header byte, a payload, and an optional CRC byte for synchronization. Although the number of preamble bytes can be programmable, the number of remaining bytes depends on the coding rate and spreading factor used in other parameters. The number of preamble symbols is generally set to *M_preamble_* = 4.25 + N_prog_, where N_prog_ is the programmed preamble length. The total number of bytes of the physical layer data frame is calculated using Equation (2) [37].

(2) $$M=[M_{preamble}+8+M_{SF}*\left(CR+4\right)]$$

(3) $$M_{SF}=max\left(\left[\frac{8PL-4SF+28+16CRC-20IH}{4\left(SF-3DE\right)}\right],0\right)$$

Equation (3) gives $M_{SF}$, which mainly gives the number of payload symbols, where $CR\in \left\{1,2,3,4\right\}$ represents the coding rate of 4/(*CR* + 4); PL is the MAC layer, including MAC header and application data payload (in bytes); *SF* is the spread spectrum factor. If the optional function *CRC* is enabled, *CRC* = 1; *IH* = 1 indicates that the implicit header function is enabled (i.e., the physical layer header is not transmitted); and *DE* = 1 indicates that the data optimization function is activated. For a given combination of spreading factor (*SF*), coding rate (*CR*), and signal bandwidth (*BW*) the total on-the-air (ToA) transmission time of a LoRa packet can be calculated using Equation (4), where *Ts* is the transmission time of one symbol, which is calculated using Equation (5).

(4) ToA=Ts∗M

(5) Ts=2SFBW

For a LoRa gateway with eight channels, Equation (6) calculates the channel capacity (i.e., number of nodes) without LBT (listen before talk) [38].

**Figure 10:**
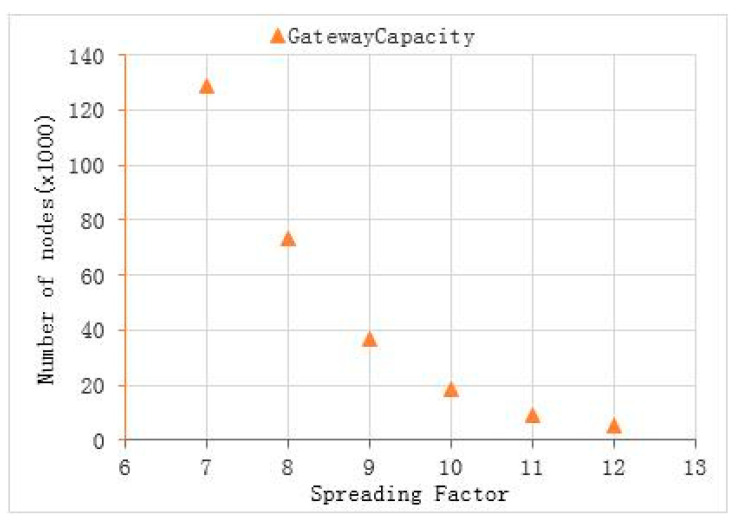
When BW = 125 kHz, T = 3600 s, the gateway capacity at different *SF*.

**Figure 11:**
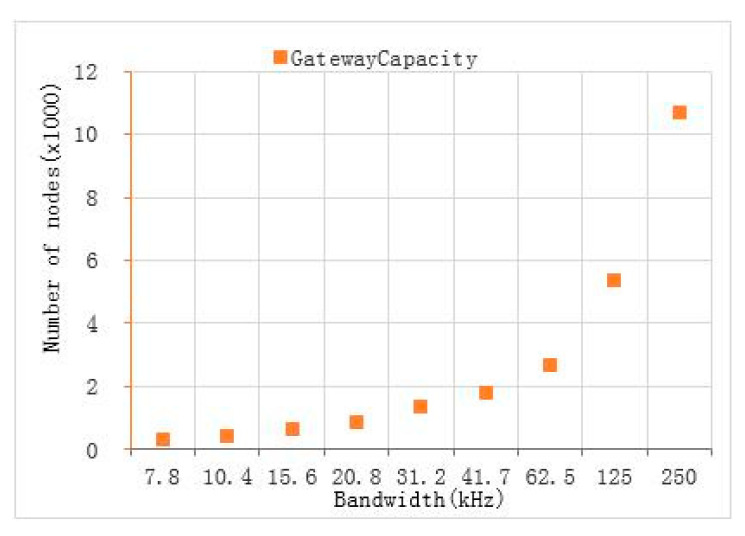
When *SF* = 12, T = 3600 s, the gateway capacity at different bandwidths.

S = 8T/(2e ∗ *ToA*) (6)

where 8 represents eight channels, T represents the transmission interval, which is related to the packet length and rate. While, ½e is the maximum throughput of the basic Aloha algorithm and e is a constant, equal to 2.718. Under the premise of 10-byte preload, the relationship between different *SF* and *BW* and their theoretical gateway capacity are shown in Figure 10 and Figure 11.

If different algorithms are adopted, this will also lead to a change of maximum throughput, resulting in a change of theoretical capacity. For example, if the precondition is modified so that each node has a LBT function and the slot Aloha algorithm is used instead of the previous basic Aloha algorithm, the maximum throughput is different, due to different algorithms. At this time, the maximum throughput is 1/e, so the theoretical capacity of the channel will be doubled. It can be seen that under the condition of setting parameters as shown in Table 3, a single LoRa gateway can theoretically connect 5345 nodes. In practical applications, the gateway can receive SF7–SF12 signal data at the same time. Due to the limited demodulation and coverage capacity of a single gateway, in reality, it is actually difficult to meet the requirements of the theoretical capacity, but it can be deployed with multiple gateways to maximize the network capacity.

### 5.3. Communication Test and Discussion

**Figure 12:**
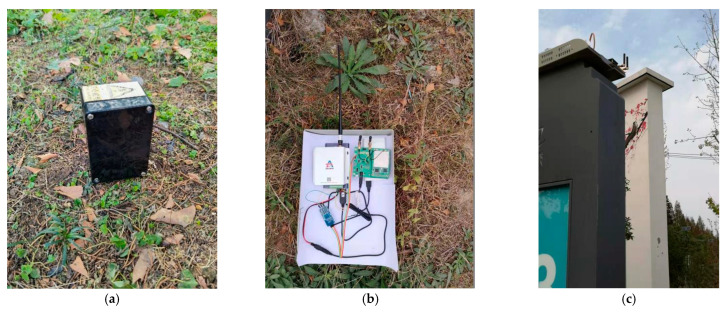
Communication test: the LoRa gateway was placed on a billboard 2.5 m above the ground. (**a**) LoRa terminal node; (**b**) LoRa gateway with the DTU; (**c**) Gateway placement location.

**Figure 13:**
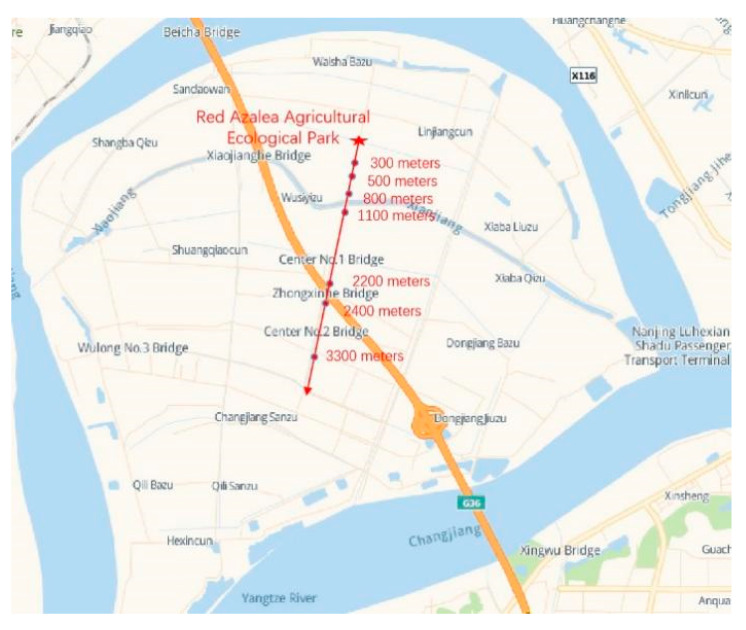
Communication test route for each designated test location, and the node used to send data in the simulations. The red star symbol indicates the starting point of the test.

In principle, a wireless communication gateway should be deployed at the highest possible position, such as a communication operator’s iron tower or the roof of a high-rise building, to improve the communication distance and signal quality. In practical applications, the site environment, operating conditions, economic cost, and other factors need to be fully considered [39]. This test took the farms around the Red Azalea Agricultural Ecological Park (RAAEP) in the Baguazhou area as the test site, to evaluate the communication distance and signal coverage between the gateway and the nodes in a typical suburban natural farmland environment. No tower or high-rise building was available for the operators in the area, no advantageous terrain was available, and certain obstacles were contained in the communication space. The test took the RAAEP as the starting point, and considering the implementation difficulty and cost control, the LoRa gateway device was deployed on a billboard approximately 2.5 m above the ground (Figure 12c), while the mobile power supply was used to power the LoRa gateway (Figure 12b). A communication test route diagram is shown in Figure 13. The AHS was specially programmed for the data transmission test as a terminal node (Figure 12a). We drove along the lane with the terminal node for the communication test, and several test points were placed in the southwest direction. Tall and dense trees were located on both sides of the road, but there were relatively open road areas at 450–500 m and 750–800 m in front of the starting point. The system started to enter a village at 1000 m, passed through the village at 1100 m, and entered woods on the two sides of the road at the same time. A highway bridge was located at 2200 m, and the test route crossed under the highway bridge.

**Figure 14:**
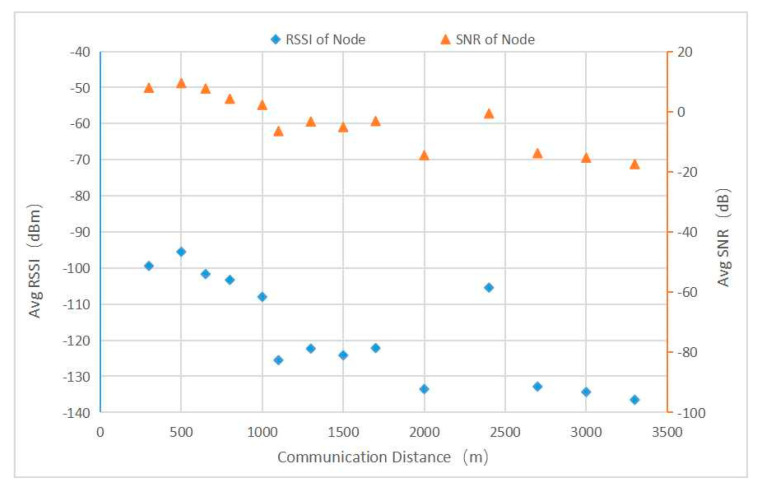
RSSI and SNR values.

At each test location, the terminal node sent a group of sequentially numbered data every 3 s, for a total of no less than 20 groups. The gateway received the data, printed the received signal strength indication (RSSI) and signal-to-noise ratio (SNR) information of the data, and uploaded this information to the cloud through a data transfer unit (DTU). We calculated the average RSSI and SNR of the test points at the same distance, which are shown in Figure 14.

**Figure 15:**
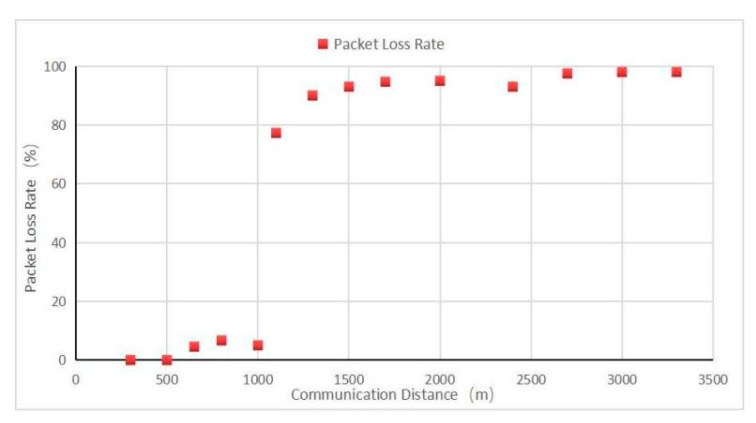
Packet loss rate.

Figure 15 shows the data packet loss rate. As seen from the figure, with the increase in the communication distance, the RSSI and SNR gradually decreased, and the packet loss rate gradually increased. At 500 m and 800 m from the test point, the area was relatively open, the influence of tree shielding was small, and the received signal improved. Although signals were still received at 1100 m, the packet loss rate was too high, and the communication accuracy was lost. Therefore, in practical applications, the communication quality evaluation should focus on more than the RSSI, and the packet loss rate was the prerequisite for the evaluation of communication quality.

In a communication system, if the signal power value in the communication link is equal to, or greater than, the sensitivity of the receiver, the receiver can normally obtain the information contained in the transmitted signal; the communication is successful. On the contrary, if the signal power is lower than the sensitivity, the quality of information obtained will be far lower than the specified requirements [40].

**Figure 16:**
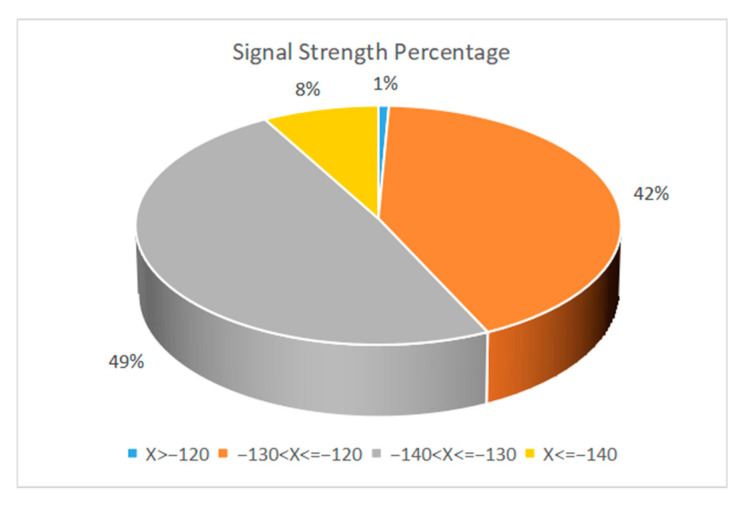
Signal strength distribution.

Figure 16 shows the obtained signal strength distribution. We selected a set of test data under relatively poor test conditions (such as antennas without enhanced gain), where the analyzed system performance would have more redundancy space. There were 135 received signal strength data points in total. During the test, the distance between the node and the gateway ranged from 300 m to 1300 m. Among the valid data points obtained, there were 18 at 300 m, 22 at 500 m, 19 at 650 m, 26 at 800 m, 19 at 1000 m, 5 at 1100 m, and 26 at 1300 m. The signal strength of these data ranged from −142.5 dBm to −119.8 dBm, including 1 data point greater than −120 dBm, 57 data points less than −120 dBm and greater than −130 dBm, 66 data points less than −130 dBm and greater than −140 dBm, and 11 data points less than or equal to −140 dBm. From the signal strength analysis, 92% of the test signals were greater than −140 dBm, while the received signal sensitivity of LoRa gateway was −142 dBm. Therefore, the RSSI of this test was within the acceptable range. However, when combined with the packet loss rate data analysis, the coverage radius of a single gateway should not exceed 1100 m.

Compared with the theoretical parameters, the actual test data parameters, especially the communication distance, had large gaps. Many factors restrict wireless communication distance.

In an ideal environment, wireless communication satisfies the Friis transmission equation [41,42]. After considering the loss of the free space path, the Friis transmission equation can be transformed into the following Equation (7):

(7) $$\mathrm{Pt}-\mathrm{Pr}+\mathrm{Gt}+\mathrm{Gr}=20\mathrm{lg}\frac{4\pi fd}{c}+Lc+L0$$

where Pt is the transmission power of the transmitter, Pr is the sensitivity of the receiver, Gt is the transmitter antenna gain, Gr is the receiver antenna gain, f is the carrier frequency, d is the distance between the receiver and transmitter antennas, c is the speed of light, Lc is the feeder loss of the transmitter antenna at the base station, and L0 is the air propagation loss. Here, π and c are constants; therefore, Equation (7) can be easily converted into the following Equation (8):

(8) $$\mathrm{Pt}-\mathrm{Pr}+\mathrm{Gt}+\mathrm{Gr}=20\mathrm{lg}\left(f\right)+20\mathrm{lg}\left(d\right)+Lc+L0-147.56\left(\mathrm{dB}\right)$$

Equation (8) can be converted to Equation (9) to calculate the distance:

(9) $$d=10^{\frac{\mathrm{Pt}-\mathrm{Pr}+\mathrm{Gt}+\mathrm{Gr}-20\mathrm{lg}\left(f\right)-Lc-L0+147.56\left(\mathrm{dB}\right)}{20}}$$

Therefore, according to the theoretical calculation formula, the factors affecting the wireless communication distance include the system’s own factors, such as receiving sensitivity, transmission power, transmitter, and receiver antenna gain, as well as environmental conditions such as obstacles, transmitter and receiver antenna height, electromagnetic interference and weather influence. In the system parameter setting designed in this paper, considering the data transmission rate and battery life, we set the maximum SF and transmission power to maximize the sensitivity of the node. Therefore, the main factors affecting the communication distance of the system came from the antenna gain and the air propagation loss caused by the obstacles between the sensor node and the gateway. In our system, we chose an antenna with high gain as much as possible; however, due to the consideration of the overall waterproof and anti-corrosion properties of the sensor node, the transmitting antenna was encapsulated inside the node, which led to increased propagation loss. As mentioned in the previous communication test section, the LoRa gateway was deployed on a billboard about 2.5 m above the ground. The sensor node test location passed through the village, and there were tall and dense trees on both sides of the test route. These test conditions well simulated the low-cost deployment mode in a typical suburban farming environment, but objectively caused the propagation loss of wireless communication and greatly reduced the wireless communication distance. Due to the implementation environmental conditions, deployment difficulty, and cost, we did not deploy the LoRa gateway at a higher position for testing, but from the calculation formula, we could show that by deploying the gateway at a commanding height over the environment, we could reduce the obstacles between communications, reduce the air propagation loss, and improve the communication distance exponentially.

Overall, when a sensor device is designed as the node of an LPWAN, the transmission power, reception sensitivity, and carrier frequency are subject to the node power consumption and chip performance factors. In practical applications, considering the implementation environmental conditions, economic cost, and deployment and maintenance difficulty, we cannot blindly pursue the ideal communication transmission distance; thus, we need to find a balance and deploy the network reasonably [43].

## 6. Conclusions

With the idea of saving resources and costs, this paper applied LPWAN technologies to an inventoried proximal soil sensor device by designing an attachment hardware system (AHS) and realized technical upgrades. Compared with conventional sensors based on wired communication technologies (such as RS-485 and SDI-12), mobile cellular technologies (such as 2G, 3G, 4G, etc.), and short-range wireless communication technologies (such as Bluetooth, ZigBee, etc.), it not only retained the original mature sensing technology of the sensor device but also exhibited ultralow power consumption and long-distance transmission, while having the advantages of an LPWAN. At experimental level, it can be seen from the actual energy consumption test and analysis that a single-battery power supply could work for more than 3 years without natural attenuation; thus, meeting the requirements of general applications. Additionally, flame-retardant epoxy resin can be used for integral molding and pouring, and this would make the system more compact as a whole, with high mechanical strength, strong heat resistance, and easy deployment, as well as being maintenance-free, waterproof, and anti-corrosion. However, traditional sensors need to be powered by mains power supply, which were troublesome to deploy in applications and required a lot of maintenance in the later stages. Even if some used a battery power supply, the sensor devices were still based on the traditional application design, and their power consumption could not use a long-term battery power supply, and were troublesome to maintain. Furthermore, through the communication distance test, signal coverage test, and gateway capacity analysis, it was shown that in a typical suburban farming environment, a single gateway could carry more than 5000 nodes within 1100 m, which could easily and quickly deploy a large-scale wireless sensor network; whereas, the traditional types would require a huge cost to achieve a large-scale sensor network. Finally, the sensor designed in this paper could obtain data remotely in real time, while the latter needed to obtain data manually on site.

The technical means to instantly obtain various soil physicochemical parameters in a field is not only an important research direction in soil science but also an important technical support tool for the development of conventional laboratory-based physicochemical soil testing and analysis procedures for field-based measurements [2]. The development and application of LPWAN technology has enabled low-cost, low-power, wide-coverage, and real-time soil field monitoring. In this paper, an AHS with LPWAN technology based on LoRa was designed and applied to an inventoried soil moisture sensor, to upgrade the technology so that it, not only retained the performance, accuracy, and reliability of the original sensor, but also had the ultralow power consumption and long-distance wireless transmission function of an LPWAN. After loading the AHS, the sensor device could be built and deployed as a node in a wireless sensor network in an economical, flexible, and convenient manner; this not only expanded the applicability of the LPWAN, but also prevented the elimination of inventoried soil moisture sensors, due to their outdated technology. It is further concluded that not only soil moisture sensors, but also other inventoried proximal soil sensor devices based on conventional communication methods (such as RS-485, SDI-12 and other data communication methods) or devices whose outputs are standard voltages or currents could be designed with, or adopt, AHSs with technical designs that require ultralow power consumption; in this way, they can not only possess the technical advantages and application capacities of an LPWAN, but also retain their original mature sensing technology and give full play to the application value and economic value of inventoried proximal soil sensor devices, to avoid a waste of resources.

## Figures and Tables

**Table 1:** The key characteristics of LPWAN technologies: Sigfox, LoRa, and NB-IoT.

	Sigfox	LoRa	NB-IoT
Frequency	Unlicensed sub-1 GHz ISM bands	Unlicensed sub-1 GHz ISM bands	Licensed LTE frequency bands
Range	10 km (urban), 40 km (rural)	5 km (urban), 20 km (rural)	1 km (urban), 10 km (rural)
Bandwidth	100 Hz	250 kHz and 125 kHz	200 kHz
Maximum data rate	100 bps	50 kbps	200 kbps
Interference immunity	Very high	Very high	Low
Adaptive data rate	No	Yes	No
Allow private network	No	Yes	No

**Table 2:** Energy consumption of each main state of the system.

	ADC Sampling Battery Voltage	Sensor Work	Data Sending and Receiving	Low Power Mode
Voltage(V)	3.3	12	3.3	3.3
Current (mA)	15.4	40.9	168.3	4.3 × 10^−3^
Duration (s)	6	3	2	T-11

**Table 3:** The key parameter settings of the node.

TFREQ	RFREQ	POW	BW	TSF	RSF	CR
475.5 MHz	506.5 MHz	20 dBm	125 kHz	12	12	4/5

**Table 4:** Range of spreading factors.

Spreading Factor	7	8	9	10	11	12
**Demodulator SNR**	−7.5 dB	−10 dB	−12.5 dB	−15 dB	−17.5 dB	−20 dB

Note that the spreading factor must be known in advance on both transmit and receive sides of the link, as different spreading factors are orthogonal to each other.

## Data Availability

Not applicable.

## Abbreviations

IoT	Internet of Things
LPWAN	Low-Power Wide-Area Network
AHS	Attachment Hardware System
ECa	apparent Electrical Conductivity
ISEs	Ion Selective Electrodes
ISFETs	Ion Sensitive Field Effect Transistors
MCU	Microcontroller Unit
PaaS	Platform as a Service
VWC	Volumetric Water Content
MQTT	Message Queuing Telemetry Transport
RAAEP	Red Azalea Agricultural Ecological Park
RSSI	Received Signal Strength Indication
SNR	Signal-to-Noise Ratio
DTU	Data Transfer Unit
ERP	Effective Radiated Power
BW	Band Width
SF	Spreading Factor
CR	Coding Rate
LBT	Listen Before Talk
